# Survey of doctors' opinions of the legalisation of physician assisted suicide

**DOI:** 10.1186/1472-6939-10-2

**Published:** 2009-03-05

**Authors:** William Lee, Annabel Price, Lauren Rayner, Matthew Hotopf

**Affiliations:** 1 King's College London, Institute of Psychiatry, Department of Psychological Medicine, 10 Cutcombe Rd, London, SE5 9RJ, UK

## Abstract

**Background:**

Assisted dying has wide support among the general population but there is evidence that those providing care for the dying may be less supportive. Senior doctors would be involved in implementing the proposed change in the law. We aimed to measure support for legalising physician assisted dying in a representative sample of senior doctors in England and Wales, and to assess any association between doctors' characteristics and level of support for a change in the law.

**Methods:**

We conducted a postal survey of 1000 consultants and general practitioners randomly selected from a commercially available database. The main outcome of interest was level of agreement with any change in the law to allow physician assisted suicide.

**Results:**

The corrected participation rate was 50%. We analysed 372 questionnaires. Respondents' views were divided: 39% were in favour of a change to the law to allow assisted suicide, 49% opposed a change and 12% neither agreed nor disagreed. Doctors who reported caring for the dying were less likely to support a change in the law. Religious belief was also associated with opposition. Gender, specialty and years in post had no significant effect.

**Conclusion:**

More senior doctors in England and Wales oppose any step towards the legalisation of assisted dying than support this. Doctors who care for the dying were more opposed. This has implications for the ease of implementation of recently proposed legislation.

## Background

Several countries or states have legislation permitting or decriminalising euthanasia or physician assisted suicide (PAS). These include Switzerland [1]; The Netherlands [2]; Belgium [3]; the US state of Oregon [4]; and, since 2008, Luxembourg [5]. These practices are legally distinct from withholding or withdrawing lifesaving or life-sustaining treatments, and from the administration of treatments which primarily aim to relieve suffering, but may incidentally also shorten life. Whilst euthanasia and PAS are supported by 70–80% of the general population in the UK [6-9], with similar proportions being found whether the research is funded by supporters of a change in the law [8,9], by detractors [7], or by independent, disinterested groups [6], the practice remains illegal.

Groups favouring a change in the law have presented opposition to euthanasia or PAS as primarily religious [10], and one independent report commented on the apparent increase in support for legalisation as an index of declining religious adherence [11]. In 2005, the British Medical Association voted narrowly to drop its longstanding opposition to assisted dying at its annual conference [12], but this opposition was reinstated the following year by a larger margin [13].

In recent years three attempts have been made to change the law in England and Wales to allow assisted dying for the terminally ill through Bills presented to Parliament by the human rights lawyer Lord Joffe [14]: the Patient (assisted dying) Bill in 2003 [15] and the Assisted Dying for the Terminally Ill Bill in 2004 and 2005 [16,17]. This proposed legislation is largely similar to the Death with Dignity Act of the US State of Oregon[4].

Under the terms of the Assisted Dying Bill, it would have become legal for doctors to prescribe a lethal dose of medication to patients who requested it, if the patient was diagnosed with a terminal illness, considered to be suffering unbearably, and had mental capacity to make the decision [18]. "Terminal illness" in this context means an illness which, in the opinion of two doctors, is inevitably progressive, cannot be reversed by treatment and will be likely to result in the patient's death within a few months [17]. The stipulation of possession of mental capacity was consistent with the decision being autonomous, and consistent with the values of the individual, with an absence of a disorder of mind or brain that could influence the decision-making process.

The Assisted Dying for the Terminally Ill Bill was defeated in the House of Lords in May 2006, but given the support for assisted dying by groups such as Dignity in Dying, and the state of public opinion, it is likely that a further Bill will be presented to Parliament in the future.

The proposed change in the law would affect the working practices of many senior doctors in England and Wales, but there are few peer-reviewed studies of their views. The most recent, published in 2006, surveyed the views of GPs in Wales. The response rate was 65%, the number responding was 1202, and 62% of these opposed a change in the law to allow physician assisted suicide [19]. In 1999 an attempt was made to survey all 742 members of the British Geriatrics Society and all 820 members of the Intensive Care Society. Participants were asked about their views on legalisation of assisted suicide and voluntary euthanasia, but only their opinions on the legalisation of active voluntary euthanasia were published. Eighty percent of geriatricians and 52% of intensive care doctors considered the deliberate administration of a treatment intended to kill as unjustified in any circumstance. The response rates were 45% and 37% in the two groups [20]. The views of the geriatricians from this survey had previously been published separately [21]. In 1998, 322 (72% participation rate) UK and Irish psychiatrists were sent a questionnaire asking their views on the legalisation of assisted suicide. Forty four percent opposed a change in the law, 18% were neutral, and 38% support this [22]. In 1994, 424 GPs and hospital consultants in one area of England were asked by questionnaire whether they thought the law in the UK should be changed to allow voluntary euthanasia, similar to the situation in the Netherlands. Of the 309 (74%) doctors who returned the questionnaire and answered the question, 146 (47%) supported a change in the law, 103 (33%) opposed any change, and 60 (20%) were undecided [23]. Three of the four surveys described above showed doctors to be more opposed than in support of changes in the law to allow assisted suicide or voluntary euthanasia. The survey which showed support for a change in the law was the earliest and concerned voluntary euthanasia rather than PAS.

There are many other (non peer-reviewed) surveys of British doctors' views in the public domain, a total of fourteen of which are thoroughly reviewed in the seventh appendix of the 2005 report of the House of Lords Select Committee on the Assisted Dying for the Terminally Ill Bill [24]. In addition to these is the submission to the committee by the Association for Palliative Medicine of a survey of 610 members carried out in 2003 showing 565 (93%) opposed legalising assisted suicide. No participation rate was published. The conclusion of the report is that, while the surveys should be interpreted with caution, doctors "...appear to be notably less in favour of legalising euthanasia [or PAS (implied)] than the general public." It is of note that a 2006 survey of doctors in the UK found low (2.6%) support for the idea that a new law to allow assisted dying or voluntary euthanasia would have helped real patients whose deaths they had attended, and a similarly low figure (4.6%) felt that the current legal situation interfered with the best management of patients [25].

There is evidence from Europe that health professionals, especially those who work with the dying, are similarly less supportive of a change in the law than the public: A Swiss survey contacted 726 palliative care specialists, 148 oncology clinicians and 140 medical students over the years 2000–2005. About a third of the members of professional groups were doctors, the rest being other healthcare professionals. The response rates were 56%, 59% and 'near 100%' respectively. The palliative care specialists were 44% in support of PAS, the oncology clinicians were 73% in favour, as were 77% of the medical students [26]. A 1998 Finnish survey attempted to compare the attitudes to PAS of 506 doctors, 800 nurses and 1000 members of the general public. The response rates were 62%, 68% and 59%. Twenty percent of the doctors, 34% of the nurses and 42% of the general public supported PAS in the scenario of an incurable cancer [27].

Even in countries where PAS is legal, support for this practice is far from universal among doctors. A Swiss group investigated the views of 2589 GPs, physicians, gynaecologists, oncologists and geriatricians in that country. The responders numbered 1650 (64%), and of them 32% had ever been asked to assist with a patient's suicide. Of these, 49.7% refused. Among those who had never been asked to assist with a suicide, 59% reported they would refuse [28].

There have been no published studies examining attitudes to PAS across all specialities and general practice in England and Wales, the region of jurisdiction of the proposed Bill. In this study we aimed to measure support for legalising physician assisted suicide, in any form, in a representative sample of senior doctors working in the NHS in England and Wales.

We found more doctors opposed than supported a change in the law to permit Physician Assisted Suicide, and that religious doctors were more likely to oppose such a change. Doctors who reported working frequently with the dying were also more likely to oppose a change in the law, but there was no effect of specialty, gender or years in post.

## Methods

We sent questionnaires to 1000 senior doctors in England and Wales randomly sampled from the Informa Healthcare Medical Directory 2005/2006 [29], a commercially available directory of medical practitioners, available on CD-ROM. In most cases the register contained each doctor's full name, address, contact telephone number and specialty. Senior doctors were defined as currently practicing as general practitioners (GPs) or on the specialist register (consultants) in any specialty. Retired doctors were excluded.

We asked those receiving the questionnaire to provide details of their specialty, how long they had been a GP or consultant, their gender and how much their day to day work involved the management of dying people. We also asked them to rate how religious they felt they were.

We provided a brief synopsis of the Assisted Dying for the Terminally Ill Bill which included the definition of the terms used in the Bill, and a clarification of what is and is not currently legal in the UK (see additional file 1), and asked doctors to what extent they supported any change in the law towards allowing physician assisted suicide to take place in England and Wales.

Questionnaires were first sent in February 2007. A second mailing to non responders was sent 12 weeks later (May 2007). We telephoned non responders after six weeks and resent questionnaires if required. On telephoning it was clear that a number of potential participants had moved, died or retired, and the denominator for the participation rate was adjusted to take account of this.

Each questionnaire was given a unique number, so that those who responded were not sent another questionnaire, but we removed all identifying information before the analysis.

We gained permission for the study from the Institute of Psychiatry, King's College London Research Ethics Committee.

The main outcome of interest was level of agreement with the statement: "The law should **not **be changed to allow assisted suicide". A secondary outcome was level of agreement with the statement, "I would be prepared to prescribe a fatal drug to a terminally ill patient who was suffering unbearably, were that course of action to become legal in the future". These were both ascertained using five-point Likert-type scales, which were then converted into three-point scales consisting of 'agree', 'neither agree nor disagree' and 'disagree' with legislation change to allow any form of physician assisted suicide, and with preparedness to carry out PAS, were it legal.

We performed separate univariable and multivariable analyses predicting the outcomes using polytomous methods. These are similar to logistic regression but they allow more than two outcomes to be predicted simultaneously. Covariates were gender, specialty, frequency of working with the dying, level of religiousness, years in post, and whether the respondent had read any of the Assisted Dying Bill. There were four relative risk ratios and confidence intervals produced for each exposure. For the main outcome these were disagreeing with any change in the law against agreeing with change and disagreeing with any change in the law against no opinion. For the secondary outcome the relative risk ratios represented not being prepared to carry out PAS were it legal against being prepared to do so, and not being prepared to carry out PAS against reporting no opinion.

## Results

**Figure 1:**
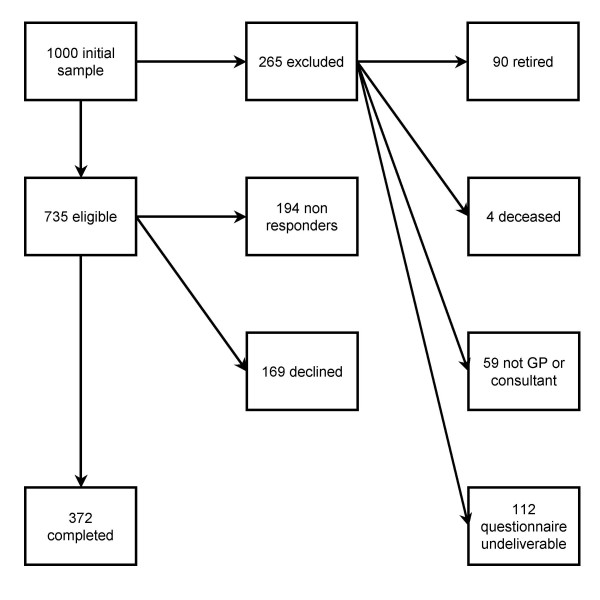
**Outcomes of the sample of senior doctors initially contacted**.

A response rate of 50% was achieved once we had accounted for exclusions (Figure 1). We found no differences between responders and non-responders (Table 1).

**Table 1:** Characteristics of sample.

	**Participants**			**Refusers**			**Non Responders**			**Total**		
	**Male**	**Female**	**Total**	**Male**	**Female**	**Total**	**Male**	**Female**	**Total**	**Male**	**Female**	**Total**
**Specialty**	**N (%)**	**N (%)**	**N (%)**	**N (%)**	**N (%)**	**N (%)**	**N (%)**	**N (%)**	**N (%)**	**N (%)**	**N (%)**	**N (%)**

**GP**	118(46)	70(60)	188(51)	65(51)	25(61)	90(53)	64(45)	39(75)	103(53)	247(47)	134(64)	381(52)
**Medical**	60(23)	30(26)	90(24)	25(20)	8(20)	33(20)	30(21)	8(15)	38(20)	115(22)	46(22)	161(22)
**Surgical**	45(18)	2(2)	47(13)	22(17)	1(2)	23(14)	29(20)	2(4)	31(16)	96(18)	5(2)	101(14)
**Psych**	18(7)	10(9)	28(8)	4(3)	3(7)	7(4)	6(4)	0(0)	6(3)	28(5)	13(6)	41(6)
**Other**	15(6)	4(3)	19(5)	10(8)	2(5)	12(7)	10(7)	2(4)	12(6)	35(7)	8(4)	43(6)
**Unknown**	0(0)	0(0)	0(0)	2(2)	2(5)	4(2)	3(2)	1(2)	4(2)	5(1)	3(1)	8(1)

**Total**	256(69)	116(31)	372(51)	128(76)	41(24)	169(23)	142(73)	52(27)	194(26)	526(72)	209(28)	735(100)

Note: Percentages within the body of the table are column percentages. Within the Total row of the table they are row percentages within supercolumns, with the exception of those also within the Total columns, which are row percentages between supercolumns. Eg 118/256=46%, 256/372=69% and 372/735=51%.

Most of the responders (93% – not shown) filled in all or nearly all of the questionnaire, leaving three or less of the 50 items blank.

Thirty-two percent of responding doctors reported having read at least some of the Bill. This did not differ by specialty (34% to 39%), except for surgical specialties, who were much less likely to have read the Bill (15%). Female doctors reported having read at least some of the Bill more frequently than male doctors (42% vs 27%).

**Figure 2:**
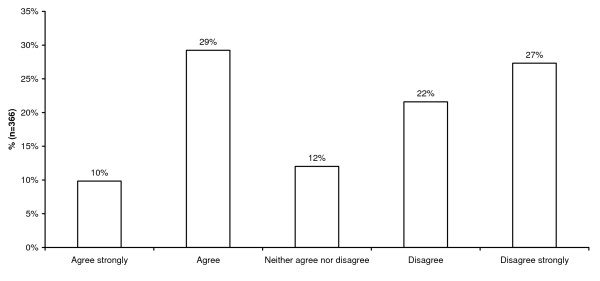
**Views of senior doctors on the legalisation of physician assisted suicide**. More doctors oppose PAS (49%) than support it (39%), and the opposition is more opposed to the Bill than the support is in favour of it.

Overall, 39% (95% CI: 34% to 44%) of the sample supported changing the law to permit PAS, 49% (44% to 54%) were opposed to a change and 12% (7% to 15%) neither agreed nor disagreed with any change. Most supporters of change in the law endorsed the option to 'agree' with some legislative step towards physician assisted suicide, whereas most doctors who were opposed to a change endorsed 'disagree strongly' (Figure 2).

Gender, specialty and years in post had no effect on support for or opposition to new law. The only significant associations were amount of time spent working with the dying, religiousness, and having read at least some of the Bill. Those doctors who spent more time caring for the dying were less likely to support a change in the law. Greater strength of religious belief was also associated with opposition to a change, as was having read at least some of the Bill (Table 2). These three variables acted independently of one another (Table 3). While less religious doctors were more likely to support a change in the law, an appreciable proportion of these (34% for "no religion" and 47% for "not very religious") were opposed to a change. A smaller proportion of religious doctors supported a change in the law (15% for "very religious" and 32% for "fairly religious").

**Table 2:** Raw data cross-tabulations of various factors with doctors views on physician assisted suicide and on whether they would assist in suicides were it legal to do so.

			**Support PAS**				**Would consider PAS if legal**			
			**Agree**	**Neither agree nor disagree**	**Disagree**	**Missing**	**Agree**	**Neither agree nor disagree**	**Disagree**	**Missing**

		N	**N (%)**	**N (%)**	**N (%)**	**N (%)**	**N (%)**	**N (%)**	**N (%)**	**N (%)**

Sex	**Male**	256	95(37)	31(12)	126(49)	4(2)	82(32)	32(13)	139(54)	3(1)
	**Female**	116	48(41)	13(11)	53(46)	2(2)	33(28)	15(13)	67(58)	1(1)
			*Chi2 = 0.65 df = 3 P = 0.89*				*Chi2 = 0.59 df = 3 P = 0.90*			

Specialty	**Consultant**	184	77(42)	21(11)	84(46)	2(1)	64(35)	29(16)	90(49)	1(0)
	**GP**	188	66(35)	23(12)	95(51)	4(2)	51(27)	18(9)	116(62)	3(2)
			*Chi2 = 2.23 df = 3 P = 0.53*				***Chi2 = 8.28 df = 3 P = 0.04***			

Care for dying	**Daily**	30	9(30)	2(7)	18(60)	1(3)	8(27)	2(7)	20(66)	0(0)
	**Weekly**	77	21(27)	9(12)	45(58)	2(3)	15(20)	7(9)	54(70)	1(1)
	**Monthly**	116	39(34)	15(13)	61(52)	1(1)	40(35)	12(10)	63(54)	1(1)
	**Yearly**	72	33(46)	12(17)	27(37)	0(0)	22(31)	15(21)	35(49)	0(0)
	**<Yearly**	37	15(41)	2(5)	18(49)	2(5)	11(30)	6(16)	20(54)	0(0)
	**Never**	35	23(66)	4(11)	8(23)	0(0)	19(54)	4(11)	11(32)	1(3)
	**Missing**	5	3(60)	0(0)	2(40)	0(0)	0(0)	1(20)	3(60)	1(20)
			***Chi2 = 32.3 df = 18 P = 0.02***				***Chi2 = 45.5 df = 18 P < 0.0005***			

Religious	**No**	110	57(51)	15(14)	38(34)	1(1)	54(49)	14(12)	43(39)	0(0)
	**Not very**	121	44(36)	18(15)	57(47)	2(2)	36(30)	23(19)	61(50)	1(1)
	**Fairly**	102	33(32)	11(11)	57(56)	1(1)	23(22)	8(8)	70(69)	1(1)
	**Very**	27	4(15)	0(0)	23(85)	0(0)	0(0)	1(4)	26(96)	0(0)
	**Missing**	11	5(46)	0(0)	4(36)	2(18)	2(18)	1(9)	6(55)	2(18)
			***Chi2 = 49.3 df = 12 P < 0.0005***				***Chi2 = 79.1 df = 12 P < 0.0005***			

Years in post	**0-**	33	11(33)	7(21)	14(43)	1(3)	7(21)	5(15)	21(64)	0(0)
	**10-**	160	66(41)	18(11)	75(47)	1(1)	50(31)	22(14)	87(54)	1(1)
	**20-**	127	39(31)	12(9)	72(57)	4(3)	36(28)	16(13)	74(58)	1(1)
	**30-**	48	25(52)	7(15)	16(33)	0(0)	22(46)	3(8)	21(44)	1(2)
	**Missing**	4	2(50)	0(0)	2(50)	0(0)	0(0)	0(0)	3(75)	1(25)
			*Chi2 = 17.4 df = 12 P = 0.14*				***Chi2 = 32.1 df = 12 P = 0.001***			

Looked at Bill	**No**	252	108(43)	34(13)	105(42)	5(2)	85(34)	36(14)	130(52)	1(0)
	**Yes**	118	34(29)	10(8)	74(63)	0(0)	30(25)	11(9)	75(64)	2(2)
	**Missing**	2	1(50)	0(0)	0(0)	1(50)	0(0)	0(0)	1(50)	1(50)
			***Chi2 = 45.7 df = 6 P < 0.0005***				***Chi2 = 52.3 df = 6 P < 0.0005***			

Support PAS	**Agree**	143	143(100)	0(0)	0(0)	0(0)	74(52)	27(19)	40(28)	2(1)
	**Neither**	44	0(0)	44(100)	0(0)	0(0)	16(36)	11(25)	17(39)	0(0)
	**Disagree**	179	0(0)	0(0)	179(100)	0(0)	24(13)	9(5)	145(81)	1(1)
	**Missing**	6	0(0)	0(0)	0(0)	6(100)	1(17)	0(0)	4(66)	1(17)
			*N/A*				***Chi2 = 114.9 df = 6 P < 0.0005***			

	**Total:**	372	143(38)	44(12)	179(48)	6(2)	115(31)	47(13)	206(55)	4(1)

Note: Tests significant at the 5% level are shown in **bold**.

**Table 3:** Associations of various factors with doctors' views on physician assisted suicide.

		**N**	**Crude Relative Risks (95% CIs)**		**Corrected Relative Risks (95% CIs)**	
			**Disagree vs Agree**	**Disagree vs Neither**	**Disagree vs Agree**	**Disagree vs Neither**
Sex	**Male**	256	1.00	1.00	1.00	1.00
	**Female**	116	0.83 (0.52 to 1.34)	1.00 (0.49 to 2.07)	0.63 (0.36 to 1.08)	0.85 (0.39 to 1.84)

Specialty	**Consultant**	184	1.00	1.00	1.00	1.00
	**GP**	188	1.32 (0.85 to 2.05)	1.03 (0.53 to 2.00)	1.03 (0.60 to 1.77)	0.74 (0.35 to 1.60)

Care for dying	**Daily**	30	1.00	1.00	1.00	1.00
	**Weekly**	77	1.07 (0.41 to 2.78)	0.56 (0.11 to 2.83)	0.92 (0.34 to 2.54)	0.47 (0.09 to 2.48)
	**Monthly**	116	0.78 (0.32 to 1.91)	0.45 (0.09 to 2.16)	0.78 (0.30 to 2.01)	0.42 (0.09 to 2.09)
	**Yearly**	72	0.41 (0.16 to 1.06)	0.25 (0.05 to 1.25)	0.38 (0.14 to 1.04)	0.22 (0.04 to 1.15)
	**<Yearly**	37	0.60 (0.21 to 1.72)	1.00 (0.13 to 7.89)	0.73 (0.24 to 2.21)	1.06 (0.13 to 8.60)
	**Never**	35	**0.17 (0.06 to 0.54)**	0.22 (0.03 to 1.47)	**0.18 (0.05 to 0.58)**	0.21 (0.03 to 1.43)
	**Missing**	5	**0.33 (0.05 to 2.37)**	Empty Cell	Empty Cell	Empty Cell
			*Test for trend: P < 0.0005*	*Test for trend: P = 0.17*	***Test for trend: P = 0.001***	*Test for trend: P = 0.20*

Religious	**No**	110	1.00	1.00	1.00	1.00
	**Not very**	121	**1.94 (1.10 to 3.43)**	1.25 (0.56 to 2.78)	**1.81 (1.00 to 3.25)**	1.23 (0.55 to 2.76)
	**Fairly**	102	**2.59 (1.43 to 4.69)**	2.05 (0.85 to 4.93)	**2.47 (1.31 to 4.65)**	1.84 (0.74 to 4.54)
	**Very**	27	**8.63 (2.76 to 26.92)**	Empty Cell	**10.01 (2.67 to 37.6)**	Empty Cell
	**Missing**	11	**1.20 (0.30 to 4.76)**	Empty Cell	0.61 (0.13 to 2.82)	Empty Cell
			*Test for trend: P < 0.0005*	*Test for trend: P < 0.004*	*Test for trend: P < 0.0005*	*Test for trend: P = 0.01*

Years in post	**0-**	33	1.00	1.00	1.00	1.00
	**10-**	160	0.89 (0.38 to 2.10)	2.08 (0.73 to 5.91)	0.84 (0.33 to 2.09)	2.15 (0.72 to 6.40)
	**20-**	127	1.45 (0.60 to 3.50)	3.00 (1.00 to 8.96)	1.29 (0.49 to 3.39)	3.25 (0.99 to 10.64)
	**30-**	48	0.50 (0.18 to 1.38)	1.14 (0.32 to 4.07)	0.59 (0.19 to 1.79)	1.37 (0.34 to 5.43)
	**Missing**	4	**0.79 (0.09 to 6.50)**	Empty Cell	Empty Cell	Empty Cell
			***Test for trend: P < 0.634***	***Test for trend: P = 0.58***	***Test for trend: P = 0.83***	*Test for trend: P = 0.47*

Looked at Bill	**No**	252	**1.00**	**1.00**	**1.00**	1.00
	**Yes**	118	2.24 (1.3 to 3.64)	2.40 (1.11 to 5.15)	**1.70 (0.99 to 2.92)**	1.80 (0.81 to 4.01)
	**Missing**	2	Empty Cell	Empty Cell	Empty Cell	Empty Cell

	**Total:**	372				

Note 1: Tests for trend exclude the rows representing missing values.

Note 2: Tests and estimates significant at the 5% level are shown in bold.

Respondents more frequently supported a change in the law (38%) than indicated they, personally, would facilitate PAS (31%) (z = 2.22 P = 0.027). There was no association between gender and being prepared to facilitate PAS. Those who worked with the dying, rated themselves as more religious, and had read at least some of the Bill were less likely to report being prepared to assist in PAS. These effects were robust to the effects of correcting for the other exposures as potential confounders (Table 4). There was some evidence that GPs were less likely to assist in PAS than hospital consultants, and that doctors who had been in post longer were more likely to be prepared to assist, but these effects were abolished when the confounders were taken into account. There was a strong, but incomplete, relationship between supporting the idea of assisted dying and being prepared to facilitate this process were it to become legal (Table 4).

**Table 4:** Associations of various factors with whether doctors would assist suicide were it legal to do so.

		**N**	**Crude Relative Risks (95% CIs)**		**Corrected Relative Risks (95% CIs)**	
			**Disagree vs Agree**	**Disagree vs Neither**	**Disagree vs Agree**	**Disagree vs Neither**
Sex	**Male**	256	1.00	1.00	1.00	1.00
	**Female**	116	1.20 (0.73 to 1.97)	1.03 (0.52 to 2.03)	0.85 (0.48 to 1.51)	0.75 (0.36 to 1.59)

Specialty	**Consultant**	184	1.00	1.00	1.00	1.00
	**GP**	188	**1.62 (1.02 to 2.56)**	**2.08 (1.08 to 3.98)**	1.39 (0.79 to 2.45)	1.60 (0.74 to 3.44)

Care for dying	**Daily**	30	1.00	1.00	1.00	1.00
	**Weekly**	77	1.44 (0.53 to 3.91)	0.77 (0.15 to 4.03)	1.24 (0.42 to 3.65)	0.71 (0.13 to 3.98)
	**Monthly**	116	0.63 (0.25 to 1.57)	0.52 (0.11 to 2.55)	0.53 (0.20 to 1.44)	0.40 (0.08 to 2.03)
	**Yearly**	72	0.64 (0.24 to 1.69)	0.23 (0.05 to 1.13)	0.60 (0.21 to 1.73)	0.20 (0.04 to 1.01)
	**<Yearly**	37	0.73 (0.24 to 2.19)	0.33 (0.06 to 1.85)	0.89 (0.27 to 2.94)	0.37 (0.06 to 2.16)
	**Never**	35	**0.23 (0.08 to 0.70)**	0.27 (0.04 to 1.75)	**0.30 (0.09 to 1.00)**	0.34 (0.05 to 2.26)
	**Missing**	5	Empty Cell	0.30 (0.02 to 4.42)	Empty Cell	Empty Cell
			*Test for trend: P = 0.001*	*Test for trend: P = 0.015*	*Test for trend: P = 0.024*	*Test for trend: P = 0.061*

Religious	**No**	110	1.00	1.00	1.00	1.00
	**Not very**	121	**2.13 (1.20 to 3.78)**	0.86 (0.40 to 1.87)	2.07 (1.14 to 3.74)	0.77 (0.35 to 1.70)
	**Fairly**	102	**3.82 (2.06 to 7.09)**	**2.85 (1.10 to 7.35)**	3.47 (1.82 to 6.64)	2.54 (0.95 to 6.76)
	**Very**	27	Empty Cell	**8.47 (1.05 to 68.2)**	Empty Cell	Empty Cell
	**Missing**	11	3.77 (0.72 to 19.61)	1.95 (0.22 to 17.65)	2.82 (0.50 to 16.0)	1.23 (0.13 to 11.93)
			*Test for trend: P < 0.0005*	*Test for trend: P = 0.002*	*Test for trend: P < 0.0005*	*Test for trend: P = 0.003*

Years in post	**0-**	33	1.00	1.00	1.00	1.00
	**10-**	160	0.58 (0.23 to 1.46)	0.94 (0.32 to 2.78)	0.46 (0.17 to 1.26)	0.77 (0.25 to 2.42)
	**20-**	127	0.69 (0.27 to 1.76)	1.10 (0.36 to 3.36)	0.44 (0.15 to 1.25)	0.66 (0.20 to 2.23)
	**30-**	48	**0.32 (0.11 to 0.90)**	1.25 (0.29 to 5.31)	0.32 (0.10 to 1.04)	1.40 (0.27 to 7.30)
	**Missing**	4	Empty Cell	Empty Cell	Empty Cell	Empty Cell
			***Test for trend: P = 0.086***	***Test for trend: P = 0.63***	***Test for trend: P = 0.10***	*Test for trend: P = 0.90*

Looked at Bill	**No**	252	**1.00**	**1.00**	**1.00**	1.00
	**Yes**	118	**1.63 (0.99 to 2.71)**	**1.89 (0.91 to 3.93)**	**1.27 (0.72 to 2.26)**	1.39 (0.63 to 3.03)
	**Missing**	2	Empty Cell	Empty Cell	Empty Cell	Empty Cell

	**Total:**	372				

Note 1: Tests for trend exclude the rows representing missing values.

Note 2: Tests and estimates significant at the 5% level are shown in bold.

## Discussion

Senior doctors are divided in their views about a change in the law to allow PAS, and fewer are in favour than are the general public in the UK [6,9]. This finding has been observed in the US [30-33], Canada [34], Finland [27] and the Netherlands [35], but this is the first survey that seeks to ascertain whether this is also true of a representative sample of senior doctors in England and Wales. Further, we found that doctors who had more day to day experience of working with the dying were more strongly opposed, as were those who rated themselves as religious.

One explanation for our findings among those who work frequently with the dying could be that it is the strong culture of palliative care in the UK which has resulted in the responding doctors being opposed to a change in the law to allow assisted suicide. There were only six respondents in the survey who reported their speciality, even in part, as palliative care and all six were opposed to a change in the law. Excluding them from the analysis however, made no substantial change to the findings (not shown).

The views of doctors who do not care for the dying are more like those of the general public, with 66% of those never caring for the dying supporting a change in the law, whilst 72% of those caring for the dying on a daily basis oppose it. This difference is not accounted for by stronger religious beliefs in those who care for the dying. Doctors who had read at least some of the Bill were more opposed to legalisation, an effect which was independent of religion and having a role in caring for the dying. It may be that greater knowledge of the proposed law influenced views, but is perhaps more likely that those most opposed take a greater interest in the debate.

Could it be that doctors' opposition to a change in the law stems from an over-optimistic belief in their ability to relieve suffering for the dying? The finding that the doctors who regularly care for the dying are more opposed than those who do not, argues against this view. It suggests instead that intimate knowledge and clinical experience of patients who are dying negatively influences views about PAS.

Fewer doctors stated that they were prepared to facilitate PAS if legalised, than were in favour of a change in the law. Some doctors who opposed any change in the law but stated they were prepared to facilitate physician assisted suicide were it to become legal, but there were more who supported a legal change but would not be prepared to carry out the act if permitted under law.

We have compared the findings of our study with surveys examining the views of the general public. Surveys of this subject are vulnerable to over- or under-estimation due to insufficient explanation of concepts and question choice which makes one answer more likely than others, meaning that their findings should not necessarily be accepted uncritically. As an example, the YouGov poll [9] is criticised for both issues because there is no explanation that an act of shortening a patients life has to be deliberately intended to kill for it to be considered physician assisted suicide within the meaning of most assisted dying laws, and the question about it asks "...do you think the law should be changed to allow [appropriate] patients to receive a prescription from their doctor to end their suffering, subject to a range of safeguards?". The similarity of estimates of support for PAS between surveys, however, does suggest that the general population seems to be more in favour of than opposed to the introduction of assisted dying legislation in the UK.

The responders and non-responders in the sample were similar on all of the criteria we were able to measure, suggesting no serious problem of response bias within the sample (Table 1). The Medical Directory claims to contain the details of more than 120,000 practicing doctors. Figures obtained from the General Medical Council GMC show approximately 250,000 doctors currently registered in the UK (Personal Communication by telephone 01/01/2006). The doctors in the sample may not be typical of doctors as a whole due to the voluntary nature of registering with a commercial provider, and the possible undesirable effects (in the form of unsolicited promotional material) of being included. Further, the database was not up to date, containing relatively few newly registered GPs or specialists (Table 2). These problems noted, we have no reason to believe this database is inferior in this regard than any other source of postal addresses of practicing UK doctors straightforwardly available to researchers. In addition, any effect of years in post was taken into account in the multivariable analyses.

The response rate of 50% was disappointing but is superior to similar surveys [20,21]. Efforts we made to boost the response rate included a personalised and individually signed letter, a repeat mailing, and personal telephone follow up [36]. Incentives such as money or gifts do marginally boost response rates, but this course was rejected because of ethical considerations and concerns over data quality. A shorter questionnaire may have resulted in a greater response rate, but this would have contained fewer data, and evidence suggests that the response rate only rises when the questionnaire is kept to a single page [36].

We suggest that qualitative research is required to understand doctors' views better. The opposition of doctors most closely involved in the care of the dying to a change in the law may pose a practical difficulty for implementing any new legislation, since under the terms of the Bill, those likely to be most involved in the process of assessment prior to assisted suicide are more opposed to a change in the law.

## Conclusion

We showed that senior doctors in the England and Wales are divided over the issue of physician assisted suicide, with more opposing than supporting any change in the law to allow this practice. This is at variance with the results of surveys of the general public which show a high and stable degree of support. Thirty one percent of the doctors questioned would be prepared to facilitate assisted suicide were it legalised, which has implications for policy makers and for those considering how this practice might be implemented were it to become law.

## Competing interests

WL, AP and MH have all experience of working in a palliative care setting. MH, WL, AP and LR do not have any religious affiliation. MH was on the Royal College of Psychiatrists working group on Assisted Dying, and during a consultation run by the College, voiced concern about a change in the law based on his experience of caring for people requesting assisted dying.

## Authors' contributions

Every task associated with this paper was carried out by one or more of the authors. The original idea for the study was by MH and WL. WL gained ethical approval, carried out the database work and drafted the questionnaire. WL and AP shared the administrative tasks associated with the mailings. Telephone follow ups were carried out by AP and WL. LR organised the returned questionnaires and carried out the data entry. WL, AP and LR carried out the analysis. AP and LR drafted the paper initially and all authors contributed to the manuscript. MH supervised all of the above processes. All authors read and approved the final manuscript.

## Pre-publication history

The pre-publication history for this paper can be accessed here:



## Supplementary Material

Additional file 1 **Appendix.** The synopsis of the Assisted Dying Bill, the definition of terms and the used questions from the questionnaire sent to participants. Click here for file
