# Supplementary material for: Survey of doctors' opinions of the legalisation of physician assisted suicide
Source: BMC Med Ethics. 2009 Mar 5;10:2. doi: 10.1186/1472-6939-10-2 (PMC2673229; doi:10.1186/1472-6939-10-2)
Supplement: Additional file 1 — Appendix. The synopsis of the Assisted Dying Bill, the definition of terms and the used questions from the questionnaire sent to participants. [file 1472-6939-10-2-S1.doc]

#
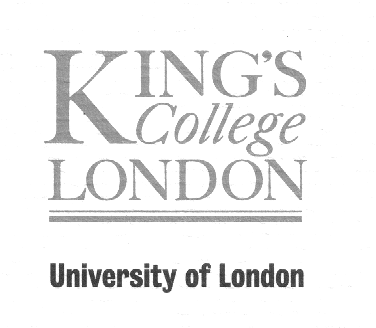


# CONFIDENTIAL

# Attitudes to the Assisted Dying for the Terminally Ill Bill among senior doctors.

- This questionnaire is part of a study asking senior doctors (GPs and Consultants) their views on Assisted Dying and Euthanasia.
- We ask that you complete the questionnaire and return it to us in the pre-paid envelope to:

# William Lee,

# Researcher in General Hospital Psychiatry

# Department of Psychological Medicine

# Institute of Psychiatry

# Weston Education Centre Cutcombe Road London SE5 9RJ

- This questionnaire will take no more than about 15 minutes to fill in.
- If you do not want to take part, please return the blank questionnaire in the pre-paid envelope so we know not to bother you again.
- The information you provide is 100% confidential.
- We welcome any questions or comments you may have.
  Please direct them to w.lee@iop.kcl.ac.uk.
- Thank you for your time.

# Attitudes to the Assisted Dying for the Terminally Ill Bill among senior doctors.

The purpose of the study is to understand current attitudes held by senior doctors about assisted dying. We are also interested in your attitudes towards a number of different but possibly related issues. All information provided by you will be treated in the strictest confidence and all data will only reported in a consolidated form, so we encourage you to be as open and honest as you can. We are independent i.e. are not funded by any organisation with a particular point of view, such as Dignity in Dying (previously Voluntary Euthanasia Society) or the Pro Life Alliance.

Thank you for completing this questionnaire.

**Respondent profile**

1a. What is your specialty? ________________________________________

1b. For how long have you been a GP or Consultant? ____________________ (years)

1c. Are you male or female? (Please circle the relevant option)

Male 1

Female 2

1d. Does your day to day work involve the management of dying people? (please circle the relevant option)

Daily 1

More often than once a week 2

More often than once a month 3

More often than once a year 4

Less often than once a year 5

Never 6

**Attitude to Assisted Suicide**

2a. Have you read the Assisted Dying Bill? (Please circle the relevant option)

No 1

Yes, partly 2

Yes 3

The Assisted Dying for the Terminally Ill Bill was, “A Bill to enable a competent adult who is suffering unbearably as a result of a terminal illness to receive medical assistance to die at his own considered and persistent request; and to make provision for a person suffering from a terminal illness to receive pain relief medication”.

Here we define the main terms used in the Bill:

- **Voluntary Euthanasia**: “ending another person’s life at his or her own request”.
- **Assisted Suicide**: “providing someone with the means to end his or her own life”.
- **Terminal illness**: “an illness which in the opinion of the consulting physician is inevitably progressive, the effects of which cannot be reversed by treatment (although treatment may be successful in relieving symptoms temporarily) and which will be likely to result in the patient’s death within a few months at most”.

Some conditions must be met before the attending physician would be able to assist a qualifying patient to die:

- Both attending and consulting physicians must be satisfied that:

(i) the patient has a terminal illness and is suffering unbearably as a result

(ii) the patient is competent to make the decision. Where this is in doubt the attending
 physician refers the patient to a psychiatrist for a psychiatric opinion to determine that the

patient is competent and not suffering from a psychiatric or psychological disorder causing

impaired judgment.

- The patient must be informed of his medical diagnosis, prognosis, the process of being assisted to die, and of the alternatives, including palliative care, hospice care and the control of pain.
- The request to be assisted to die must come from an informed decision and be made voluntarily.
- Physicians are not required to participate in any diagnosis, treatment or other action authorised by the Act to which he or she has a conscientious objection.

*Note: The Attorney-General has confirmed that it is not murder “where a doctor acts to do all that is proper and necessary to relieve pain with the incidental effect that this will shorten a patient’s life”*

*(The Assisted Dying for the Terminal Ill Bill Report).*

2b. To what extent do you agree with this statement?

NB: Assisted Suicide is defined in the Bill as “providing someone with the means to end his or her own life”

| The law should **not** be changed to allow assisted suicide. | (please circle the most appropriate response) | | | | |
| --- | --- | --- | --- | --- | --- |
|  | 1 | 2 | 3 | 4 | 5 |
|  | Agree Strongly | Agree | Neither agree nor disagree | Disagree | Disagree strongly |

2c: To what extent do you agree with this statement?

| I would be prepared to prescribe and administer a fatal drug to a terminally ill patient who was suffering unbearably, were that course of action to become legal in the future. | (please circle the most appropriate response) | | | | |
| --- | --- | --- | --- | --- | --- |
|  | 1 | 2 | 3 | 4 | 5 |
|  | Agree Strongly | Agree | Neither agree nor disagree | Disagree | Disagree strongly |

**Religious beliefs and practice**

We are interested in your religious belief and affiliation and its possible influence on your attitudes to Voluntary Euthanasia and Assisted Suicide.

4d. Please answer the question below.

| How religious would you say you are? | (please circle the most appropriate response) | | | |
| --- | --- | --- | --- | --- |
|  | 1 | 2 | 3 | 4 |
|  | Not Religious at All | Not very Religious | Fairly Religious | Very Religious |

**Thank you very much for taking part in this study.**

**Now please send the questionnaire back to us in the postage paid envelope.**
